# Finger Tapping Clinimetric Score Prediction in Parkinson's Disease Using Low-Cost Accelerometers

**DOI:** 10.1155/2013/717853

**Published:** 2013-04-16

**Authors:** Julien Stamatakis, Jérome Ambroise, Julien Crémers, Hoda Sharei, Valérie Delvaux, Benoit Macq, Gaëtan Garraux

**Affiliations:** ^1^Movere Group, Cyclotron Research Centre, University of Liege, Sart Tilman B30, 4000 Liège, Belgium; ^2^Institute of Information and Communication Technologies, Electronics and Applied Mathematics, Université Catholique de Louvain, Place du Levant 2, 1348 Louvain-la-Neuve, Belgium; ^3^Institut de Recherche Exprimentale et Clinique, Center for Applied Molecular Technologies, Université Catholique de Louvain, Chapelle-aux-Champs 30, 1200 Woluwé-St-Lambert, Belgium; ^4^Department of Neurology, University Hospital Centre, University of Liege, Sart Tilman B35, 4000 Liège, Belgium

## Abstract

The motor clinical hallmarks of Parkinson's disease (PD) are usually quantified by physicians using validated clinimetric scales such as the Unified Parkinson's Disease Rating Scale (MDS-UPDRS). However, clinical ratings are prone to subjectivity and inter-rater variability. The PD medical community is therefore looking for a simple, inexpensive, and objective rating method. As a first step towards this goal, a triaxial accelerometer-based system was used in a sample of 36 PD patients and 10 age-matched controls as they performed the MDS-UPDRS finger tapping (FT) task. First, raw signals were epoched to isolate the successive single FT movements. Next, eighteen FT task movement features were extracted, depicting MDS-UPDRS features and accelerometer specific features. An ordinal logistic regression model and a greedy backward algorithm were used to identify the most relevant features in the prediction of MDS-UPDRS FT scores, given by 3 specialists in movement disorders (SMDs). The Goodman-Kruskal Gamma index obtained (0.961), depicting the predictive performance of the model, is similar to those obtained between the individual scores given by the SMD (0.870 to 0.970). The automatic prediction of MDS-UPDRS scores using the proposed system may be valuable in clinical trials designed to evaluate and modify motor disability in PD patients.

## 1. Introduction

The most important functional disturbance in patients with Parkinson's disease (PD), a chronic neurodegenerative condition, is a disorder of voluntary movement prominently characterized by slowness. This phenomenon is generally called bradykinesia [1]. Tremor and muscle rigidity are also part of the motor phenotypic spectrum [2]. Although it has not been possible to define a single underlying pathophysiologic mechanism that explains everything, bradykinesia and other motor symptoms seem to be related to a progressive loss of dopaminergic neurons in the substantia nigra [2, 3].

Since decades, the medical community has been developing clinical tools such as rating scales to quantify the severity of motor and other symptoms in PD. Despite the various attempts to use instruments and devices for quantification, clinical scales remain the preferred method because they are easy to administer and widely available.

In the late eighties, the Unified Parkinson's Disease Rating Scale (UPDRS) was proposed as the primary international rating scale for PD clinical care and research and is still anchored in the daily practice of MDs. The motor examination part of the UPDRS requires the Specialists in Movement Disorders (SMDs) to score motor disturbances on a 5-point scale ranging from 0 (normal performance) to 4 (severe, unable to perform the task) on the basis of visual inspection. In 2008, the Movement Disorder Society has published an upgraded version of the original UPDRS [4], based on the critiques that were formulated by the Task Force for Rating Scales in Parkinson's disease [5]. The new scale (MDS-UPDRS) has been shown to be more sensitive for slight impairments and to enable a more objective rating through detailed instructions for all tasks. However, the MDS-UPDRS still suffers from methodological limitations common to all clinical rating scales including subjectivity and interrater variability [6].

Objective quantitative measures using movement sensors can assist clinicians in evaluating motor deficits. Inertial and magnetic sensors have been proposed to quantify motor performances in various medical applications [7], from the distinction between normal and pathological walking patterns [8] to the estimation of upper limb orientation based on accelerometer and gyroscope measurements [9]. These sensors are small, low-cost, light-weighted, and can record components of the movements as accelerations or displacements. Their use is not restricted to laboratory environments [8], and they do not suffer from occlusion problems as expensive visual marker-based tracking systems [10]. Accelerometers have therefore become a preferred choice for continuous, unobtrusive, and reliable method in human movement quantification [7]. In PD, inertial sensors have been proposed to investigate the asymmetry of tremor intensity and frequency [11], to quantify tremor and bradykinesia [12], to study the dynamics of resting and postural limb tremor [13], or to analyze the dynamic voluntary muscle contractions [14]. Accelerometers have also been used to quantify the impairment of finger tapping (FT) movements [1, 15, 16], to study the effect of movement frequency on repetitive finger movements [17], or to propose new parameters for the quantification of the FT test [18].

The reason why those more sophisticated sensor systems have not been adopted yet in daily clinical practices is that those systems are too expensive, too sophisticated for clinical needs, and too heavy to handle for patients. A way to improve movement evaluation while still using clinical scales is to develop systems that will assess movements during clinical scale tasks and predict clinical scale scores automatically. A first approach consists in evaluating the correlations between kinematic measures from motion sensors and clinical scales, which is a growing field of research. The reliability of a Modified Bradykinesia Rating Scale and its correlation with kinematic measures from inertial sensors has recently been evaluated [19]. Giuffrida et al. have evaluated the correlation between a multiple linear regression model and original-UPDRS scores for tremor tasks [6].

In order to improve the diagnosis accuracy of PD, a tool to predict FT scores from MDS-UPDRS FT task movement features is presented here.

## 2. Materials and Methods

### 2.1. Subjects and Materials

Thirty-six PD patients (mean age ± SD = 63.9 ± 9.1 year, range 37–79; 28 males; mean disease duration = 7.5 ± 4.0 year, total score of MDS-UPDRS motor examination = 32 ± 10.8) and ten healthy volunteers (mean age ± SD = 59 ± 15.2 year, range 38–87; 5 males) participated in the study to create a set of observations with a range of FT scores between 0 and 4, as rated by three SMDs, according to the MDS-UPDRS criteria (Table 1). PD was diagnosed according to the UK Parkinson's Disease Society Brain Bank criteria [20]. The on/off medication status was not taken into consideration and is irrelevant for the purpose of the present study. Subjects were recruited at the Cyclotron Research Centre and at the Department of Neurology, University Hospital Centre, Liege, Belgium. All patients provided written informed consent. This research protocol has been approved by the local ethical committee.

**Figure 1:**
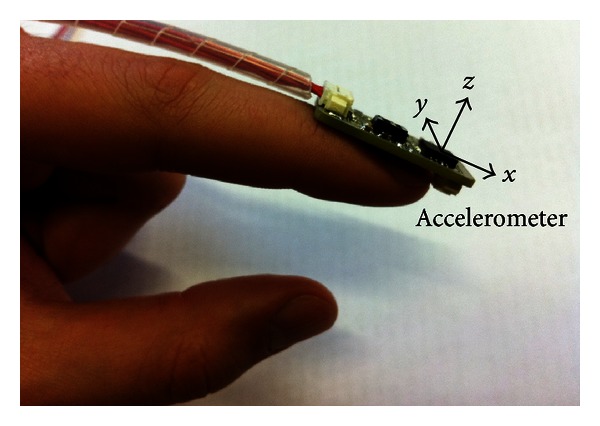
The accelerometers were placed on the tip of the index finger of both hands. The *z*-axis of the accelerometer is vertically perpendicular to the index finger, the *x*-axis is parallel, and the *y*-axis is horizontally perpendicular.

The 3-axis accelerometers recording accelerations up to ±10 g (1 g = 9.81 m/s^2^) were placed on the tip of the index finger of both hands. The *z*-axis of the accelerometers was vertically perpendicular to the index finger axis, the *x*-axis was parallel, and the *y*-axis was horizontally perpendicular, as presented in Figure 1. Accelerometers have been calibrated using a minimization function based on the norm and direction of the gravity field [21]. Data were recorded at the sampling frequency of 167 Hz and analyzed using Matlab 7.6.0 (MathWorks, Natick, MA, USA).

All participants were administered the motor examination (part III) of the MDS-UPDRS. The instructions were clearly explained and demonstrated to the subjects before performing the task, according to the MDS-UPDRS instructions. In the FT task, subjects were asked to tap the index finger on the thumb 10 times as quickly and as big as possible. If the patient did not stop, the examiner provided a stop signal and only the first 10 movements were taken into account for subsequent analysis. Subjects were instructed to start with open fingers, at maximum amplitude. Each hand was tested separately for the 46 subjects, leading to 92 observations. Three patients presented a score of 4 on both hands. As by definition they were not able to perform the task, and their observations were removed for further analysis, leading to a remaining set of 86 observations. The system was immediately able to qualify the 6 excluded observations as scores 4 (see Section 3.1). Each observation was video recorded to allow scoring by three SMDs, according to the MDS-UPDRS instructions presented in Table 1 [4]. For each observation, the SMD consensus score was defined as the mean score rounded to the nearest integer.

### 2.2. Signal Epoching

**Figure 2:**
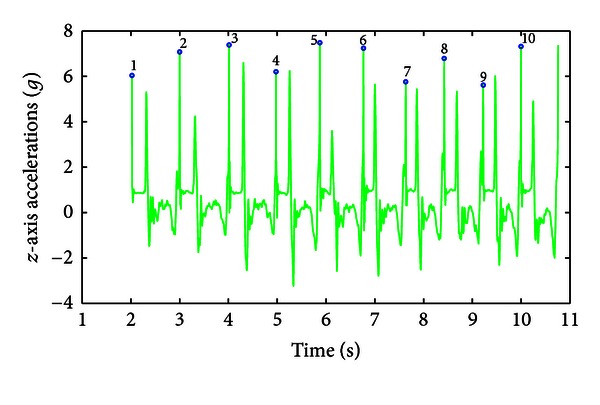
*z*-axis raw signal for an SMD consensus score of 0 obtained from a healthy volunteer performing 10 FT movements. The bullets topped by numbers represent the beginning of a new tapping movement, that is, when the index taps the thumb. The peak in the midst of each movement depicts the finger opening phase.

**Figure 3:**
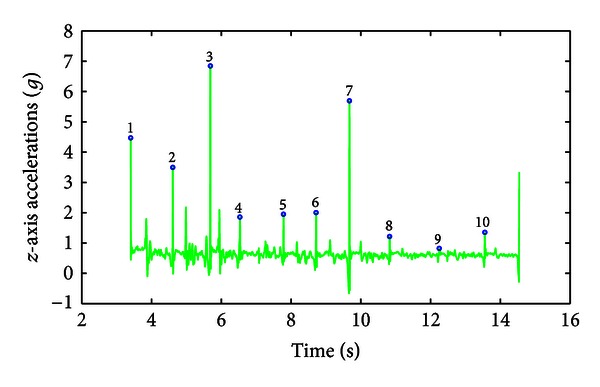
*z*-axis raw signal for an SMD consensus score of 3 obtained from a PD patient performing 10 FT movements. The bullets topped by numbers represent the beginning of a new tapping movement, that is, when the index taps the thumb. The peak in the midst of each movement depicts the finger opening phase.

The first processing step was to epoch the recorded data in order to isolate the first 10 consecutive FT movements or samples. A single FT movement was defined as the interval between two taps, that is, when the index tapped the thumb, which resulted in a high frequency and high amplitude peak in the *z*-axis signal. These peaks were identified using the high frequency output of the Daubechies wavelet transform (*db4*) of the recorded *z*-axis signal. The *z*-axis recorded accelerations and their epoching are presented for an SMD consensus score of 0 obtained in a healthy volunteer (Figure 2) and for an SMD consensus score of 3 obtained in a PD patient (Figure 3). Another important landmark in each FT movement is the time for maximum opening acceleration that is represented by a low frequency but high amplitude peak in the midst of the *z*-axis movement, which occurs when the subject splits off his/her fingers. 

### 2.3. Feature Definition and Extraction

According to the MDS-UPDRS, bradykinesia during the FT task is clinically characterized by movement slowness, and/or a decrementing speed and/or amplitude of repetitive movements, and/or hesitations [22]. Hesitations can occur at the initiation of the opening/closing movement (initiation hesitation) or during the finger opening movement (execution hesitation or hypometria).

After epoching, 8 MDS-UPDRS features were defined from the 10 consecutive FT movements to capture these clinical characteristics on the basis of the computed movement frequency, the opening angle, the level of hypometria, and their linear changes across the 10 FT movements. Ten accelerometer-specific features were also extracted on the basis of the percentage of movement time for maximum opening acceleration, maximum closing and opening accelerations, and their linear changes across the 10 FT movements.  Table 2 gives a summary of the features along with their minimum and maximum values across the 86 observations.

Among the MDS-UPDRS features, the movement speed was expressed through the mean movement frequency (*Freq*) which was computed from the inverse of each movement time. In order to depict the prospective decrementing speed (*Dfreq*), we estimated the number of movements executed before the decrement started using a statistical *t-*test. For each FT movement in a given series, we compared the mean FT frequency of the remaining movements with the mean frequency of FT movements already completed. We examined whether this difference was significant using a *t-*test (*P* < 0.05). If the difference in frequency for two consecutive *t-*tests was significant, the FT movement leading to the first significant difference was used to determine the prospective decrementing speed onset. Therefore, if no decrement was observed, a value of 10 was obtained for that feature. The same method was used to detect an increase in movement speed (*Afreq*).

The possible presence of halts in the movement (*Halts*) was also a clinical characteristic. Halts were detected if the difference between a movement frequency and the frequency slope (linear changes among the 10 samples, computed with the *robustfit* Matlab function) was above a given threshold.

The number of hesitations was also computed (*Hesits*). A sample was defined as a hesitation if its frequency or percentage movement time for maximum opening acceleration was outside a range defined from the mean, the standard deviation, and the slope. Each sample with a hesitation increments the value of the *Hesitation* feature.

The *level of hypometria* can be depicted by the smoothness of the opening movement (*Hypom*). For a healthy subject, this movement is composed of one acceleration and one deceleration, which gives the lower frequency but high amplitude peak in the midst of the FT movement. In PD patients, this opening movement could be a mix of multiple accelerations and decelerations, due to execution hesitations, which are reflected by multiple peaks in the recorded *z*-axis acceleration. Indeed, bradykinesia is characterized by the inability to energize the appropriate muscles to initiate and maintain large and fast movements. PD patients therefore need series of multiple agonist bursts to accomplish a larger movement [22]. Here, these bursts were detected using the *findpeaks* Matlab function. Each sample with more than two peaks was used to increment the value of the *Hypometria* feature.

In order to express the amplitude of the movement (*Angle*) and its possible decrementation (*Dangle*), the mean of the opening angles was computed. An attribute of accelerometers is that static acceleration due to gravity is recorded as well as inertial components of movements [8]. Under static or quasistatic conditions, that is, when the recorded acceleration is mainly due to gravity, the accelerometer can be used as an inclinometer and basic trigonometry gives the angle of tilt. The opening angle is based on the value of the gravity in the *x*-axis when the fingers are open (*a*_2_^*x*^) and closed (*a*_1_^*x*^), according to (1). During these two periods, the recorded acceleration is only due to gravity and tremor. As tremor is minimum in the *x*-axis, the parallel direction to the finger, the mean gravity is computed on that axis. The detection of open and closed fingers is based on the variance of the *x*-axis processed signal. In order to depict the prospective decrementing amplitude, the same *t-*test method as presented before is used. Consider

(1) opening angle=θopen−θclosed=arcsin(a2x)−arcsin(a1x).

Among the accelerometer-specific features, we defined the mean (*Topen*), standard deviation (*sdTopen*), and slope (*slTopen*) of the percentage of movement time when maximum opening acceleration occurred across the 10 FT movements.

The mean, standard deviation, and slope of maximum closing accelerations (*Aclose*, *sdAclose,* and *slAclose*) and maximum opening accelerations (*Aopen*, *sdAopen,* and *slAopen*) were also extracted from the *z*-axis recorded accelerations. The features associated with the maximum closing acceleration express the strength of the finger tap while the features associated with the maximum opening acceleration represent the speed of the opening movement. Since the *Aclose* and *Aopen* features are based on the amplitude of the accelerations, the gravitational artifact due to the gravity component in the recorded acceleration must be considered. The DC component of gravitational acceleration can easily be removed by high-pass filtering. However, the task of separating the gravitational and inertial components of acceleration at the frequency of rotation is impossible unless multiple sensors are used [23]. As feature extraction is performed, the gravitational artifact is no longer a problem as long as it remains constant or negligible as compared with the range of the feature. When the fingers are closed, the gravity field is parallel to the *z*-axis and its value is about 1 g. As the subject splits off its fingers, the *z*-axis component of the gravity varies as *g* cos⁡*α*, where *α* is the angle between the *z*-axis and the gravity field. The maximum closing acceleration is extracted as the peak acceleration when the index taps the thumb, that is, when the *z*-axis is parallel to the gravity field, according to the instructions given to the subjects. Therefore, the gravitational artifact is nearly constant at this time. The maximum opening acceleration occurs at the beginning of the opening movement, that is, when *α* ≤ 45°. The gravitational artifact has then a maximum variation of 0.292 g, which is negligible as compared with the range of the feature value (9.538 g—Table 2).

The last extracted feature was the root mean square (RMS) that gives a measure of the signal magnitude, as used in [6].

Altogether, the MDS-UPDRS features and the accelerometer-specific features formed a set of 18 FT task movement features.

### 2.4. Construction of the Predictive Model

The main goal of this work was to develop a tool to predict objective MDS-UPDRS scores from FT task movement features and to identify which of these features best predicted MDS-UPDRS FT scores given by 3 SMDs independently, on the basis of the corresponding video recordings. As the outcome is discrete and has a natural order, the MDS-UPDRS score prediction problem was addressed using an ordinal logistic regression model.

#### 2.4.1. Ordinal Logistic Regression Model

Logistic regression is a statistical tool used to predict a discrete outcome, such as group membership, from a set of predictor variables that may be continuous or discrete. If the outcome is binary (*y* = 0,1) and if we have *p* predictor variables *x*_1_, *x*_2_,…, *x*_*p*_, the systematic part of the binary logistic regression model is defined as follows:

(2) $$\begin{array}{c} \log\left(\theta \right)=\alpha +\beta_{1}x_{1}+\beta_{2}x_{2}+\cdots +\beta_{p}x_{p}, \end{array}$$

with

(3) $$\begin{array}{c} \theta =\frac{P\left(y=1\;|\;x_{1},x_{2},\ldots ,x_{p}\right)}{1-P\left(y=1\;|\;x_{1},x_{2},\ldots ,x_{p}\right)}. \end{array}$$

The logit of the probability of the outcome *y* (i.e., the logarithm of the odds *θ* of event *y*) is modeled as a linear combination of the predictor variables. When the outcome is discrete but not binary as in this study (*y* = 0, 1, 2 or 3), the binary logistic regression model can be extended into an ordinal logistic regression model, by taking into account the ordinal nature of the outcome. Here, the probability that a subject belongs to one of the categories equal or ordered before *j* (*P*(*y* ≤ *j*)) is compared to the probability that the patient belongs to one of the categories ordered after *j* (*P*(*y* > *j*)). The systematic part of the ordinal logistic regression model is defined as follows:

(4) $$\begin{array}{c} \log\left(\theta_{j}\right)=\alpha_{j}-\beta_{1}x_{1}-\beta_{2}x_{2}-\cdots -\beta_{p}x_{p}, \end{array}$$

with

(5) $$\begin{array}{c} \theta_{j}=\frac{P\left(y\leq j\;|\;x_{1},x_{2},\ldots ,x_{p}\right)}{P\left(y>j\;|\;x_{1},x_{2},\ldots ,x_{p}\right)}\;\text{with}\;\left(j=0,1,2\right). \end{array}$$

The *α*_*j*_ and *β* coefficients are estimated from the data by using a maximum likelihood procedure. In these equations, we observe that each of the three logit log⁡(*θ*_*j*_) has its own *α*_*j*_ term but the same *β* coefficients. It means that the effect of the predictor variables is the same for the three logit or, equivalently, for each odds *θ*_*j*_. We also observe a minus sign before the predictor variables coefficients because probabilities in the ordinal logistic regression model are defined in a different way from those in the binary logistic regression model. A positive estimation of a parameter therefore indicates a positive correlation between its associated variable and the SMD consensus score.

In order to use this ordinal regression model as a prediction tool, the model must first be trained on a training dataset. In this training dataset, each observation is associated with the FT task movement features (considered as predictor variables in the model) as well as an SMD consensus score (considered as the outcome variable in the model). Then, the ordinal logistic regression model can be applied to new observations with unknown MDS-UPDRS scores in order to predict these scores from their FT task movement features. The probability to belong to each ordered class *P*(*y*) can indeed be computed for a single observation from the *θ*_*j*_ values. Then, a continuous score can be obtained by summing the values of the outcome (0, 1, 2, or 3) multiplied by the estimated *P*(*y*). This continuous score ranges between 0 and 3 and can be discretized, if necessary, by using thresholds. 

**Figure 4:**
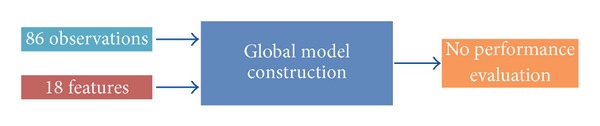
A global model was trained based on the 86 observations, each one of them being associated with 18 features as well as one SMD consensus score. Since all the observations were used for the construction of the global model, the predictive performance of the model could not be evaluated on an independent dataset.

**Figure 5:**
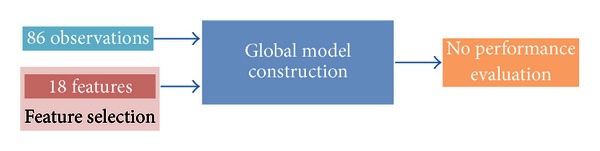
Not all 18 features may be useful in building the model. A subset of features can be selected to maximize the predictive performance of the model.

**Figure 6:**
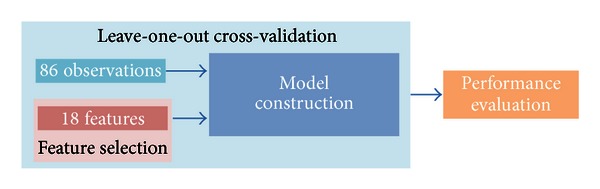
In order to evaluate the predictive performance of the global model, a leave-one-out cross-validation approach was used.

 A global model was trained on the 86 observations (Figure 4). It is of note that all eighteen features may not be useful in building the model. A subset of features can be selected to try to maximize the predictive performance of the model. Here, we used a greedy backward algorithm to select the subset of features that best predicted MDS-UPDRS FT scores (Figure 5). Then, since the global model was trained on all the observations, its predictive performance could not be evaluated on an independent dataset. Therefore, in order to estimate the predictive performance of the global model, a leave-one-out cross-validation approach was used (Figure 6). Construction of the global model, feature selection, and performance evaluation were performed using the *Design*, *vcdExtra,* and *Zelig* R packages (The R Project for Statistical Computing).

#### 2.4.2. Feature Selection

For the global model construction, feature selection was performed once on the 86 observations. Indeed, a subset of the 18 features extracted from the FT task only could be useful to build the ordinal logistic regression model in order to maximize the predictive performance of the model. The selection of the variable set maximizing the predictive performance of the model is known as a variable selection problem [24]. In order to take into account the interaction effect between the predictor variables, we used a wrapper technique for variable selection. Various subsets of variables, that is, features, are generated and evaluated [25]. The various subsets of features are generated using a greedy backward selection. Greedy search strategies are computationally advantageous and robust against overfitting [24]. The idea is to start with a model containing all the features and to evaluate its predictive performance. Then, the less relevant features are removed iteratively. The predictive performance associated to a subset of features was evaluated in two steps. First, an inner cross-validation loop was performed in order to obtain a prediction score for each observation. Second, the Goodman-Kruskal Gamma index between the predictions and the SMD consensus scores was computed and defined as the performance criterion. This index tests the strength of association of cross-tabulated data when both variables are measured at the ordinal level [26]. At each iteration, the feature elimination conducting to the highest improvement of the Goodman-Kruskal Gamma index was performed. The backward elimination stops when any feature elimination leads to a decrease of this index.

#### 2.4.3. Predictive Performance Evaluation

Leave-one-out cross-validation, with an inner and outer loop, was performed to estimate the predictive performance of the global model. At each iteration of the outer loop, the dataset was separated into a learning part and a test part. The learning part contained 85 observations while the test part only contained the remaining observation. The learning part was used to select the relevant predictor variables through a wrapper technique (using an inner cross-validation loop as explained in Section 2.4.2) and to train the ordinal logistic regression model. The selection of the relevant variables was performed only on the training part in order to avoid overestimation of predictive performance. After the training step, the model was used to predict the MDS-UPDRS score of the remaining observation in the test part. This procedure has been repeated 86 times in order to have predictions for all the observations. Eighty-six subsets of relevant features were therefore obtained during cross-validation. Finally, each observation was associated to a continuous prediction between 0 and 3 as well as a discrete SMD consensus score (0, 1, 2, or 3). Various performance indexes were computed in order to evaluate the predictive performance of the model.

The area under the curve (AUC) of the receiver operating characteristic (ROC) was computed. The ROC curve plots the sensitivity (true positive rate) against 1− specificity (false positive rate) for consecutive thresholds used to define predicted positives and negatives from the continuous scores [27]. An AUC of 0.5 corresponds to a noninformative model while an AUC of 1 corresponds to a perfect model. The accuracy was defined as the proportion of correct classifications among all the classifications. Sensitivity, specificity, and consequently AUC, as well as accuracy, can only be computed for binary classification tasks. As the outcome is discrete with four ordered classes, the problem had to be reformulated into the three following binary classification problems:

- first classification task: separate observations with a score of 0 from observations with a score greater than 0;
- second classification task: separate observations with a score of 0 or 1 from observations with a score greater than 1;
- third classification task: separate observations with a score of 0, 1 or 2 from observations with a score greater than 2.

In order to compute the next performance indexes, discretized scores were necessary. Thresholds were used to discretize the continuous scores into the four ordered classes. The sensitivity, specificity, and accuracy defined in (6) were then computed as follows:

(6) $$\begin{array}{c} \text{sensitivity}=\frac{\text{TP}}{\text{TP}+\text{FN}},\\ \text{specificity}=\frac{\text{TN}}{\text{TN}+\text{FP}},\\ \text{accuracy}=\frac{\text{TP}+\text{TN}}{\text{TP}+\text{TN}+\text{FP}+\text{FN}}, \end{array}$$

where TP, TN, FP, and FN denote the number of true positives, true negatives, false positives, and false negatives, respectively.

The discretized predictions were also used to compute the Goodman-Kruskal Gamma index between the predictions and the SMD consensus scores. The values of Goodman-Kruskal Gamma index range from −1 (negative association) to 1 (perfect agreement).

## 3. Results

### 3.1. SMD Consensus Scores

According to the SMD consensus scores, 12 observations led to a score of 0, 32 observations led to a score of 1, 31 observations led to a score of 2, and 11 observations led to a score of 3. Three patients also obtained a score of 4 in both hands. However, those patients were not included in the analysis since they could not perform the task. No movement features could be extracted from their movements and the system immediately gave them a score of 4 based on the low variance of the recorded signals, with a 100% accuracy. The Goodman-Kruskal Gamma indexes obtained between the individual scores given by the three SMD and the SMD consensus scores are 0.922, 0.982, and 0.992, respectively. The Goodman-Kruskal Gamma index obtained between the individual SMD scores varies between 0.870 and 0.970.

### 3.2. Signal Epoching and Feature Extraction

Visual inspection of epoched signals showed a very high accuracy (99%). Two parameters expressing the position and the width of the peak detection windows had to be adapted for the epoching of signals recorded on tremulous PD patients and for those with a score of 3, who typically have hesitations or halts in movements.

**Figure 7:**
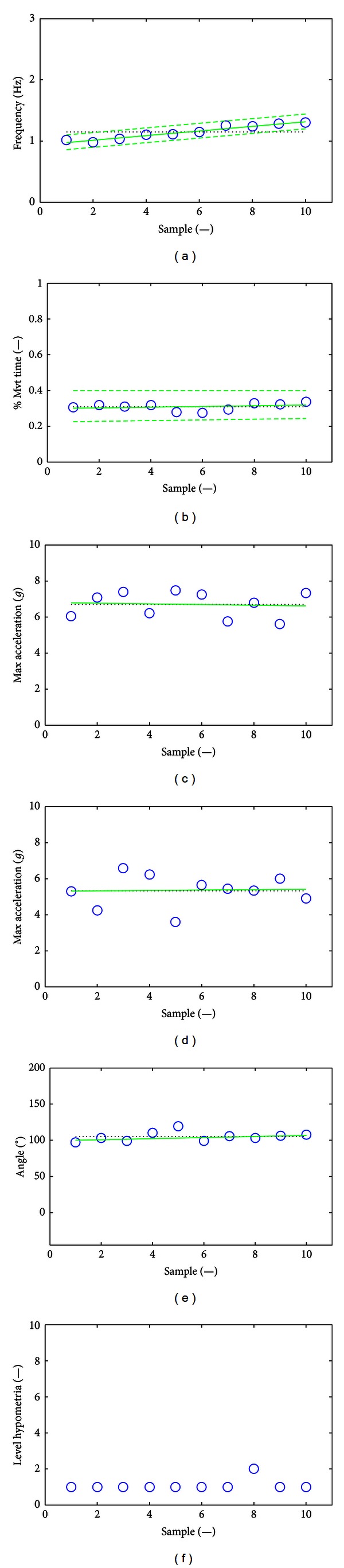
Typical features for an SMD consensus score of 0. Plot (a) gives the movement frequencies (circles). Plot (b) gives the percentages of movement time for maximum opening acceleration. Plot (c) gives the maximum closing accelerations. Plot (d) gives the maximum opening accelerations. Plot (e) gives the opening angles. Plot (f) gives the level of hypometria (circles) and the possible presence of initiation hesitations (triangles). For each plot except (f), the mean is in dotted line, the linear regression among points is in continuous line, and the limits for the detection of hesitations are in dashed lines (plots (a) and (b) only). The healthy volunteer performed repetitive FT movements at a low and slowly growing frequency and did not have any hesitations or halts. The percentage time for maximum opening acceleration is steady over samples, as maximum closing and opening accelerations. The movement amplitude is big and constant, showing no decrementing amplitude.

**Figure 8:**
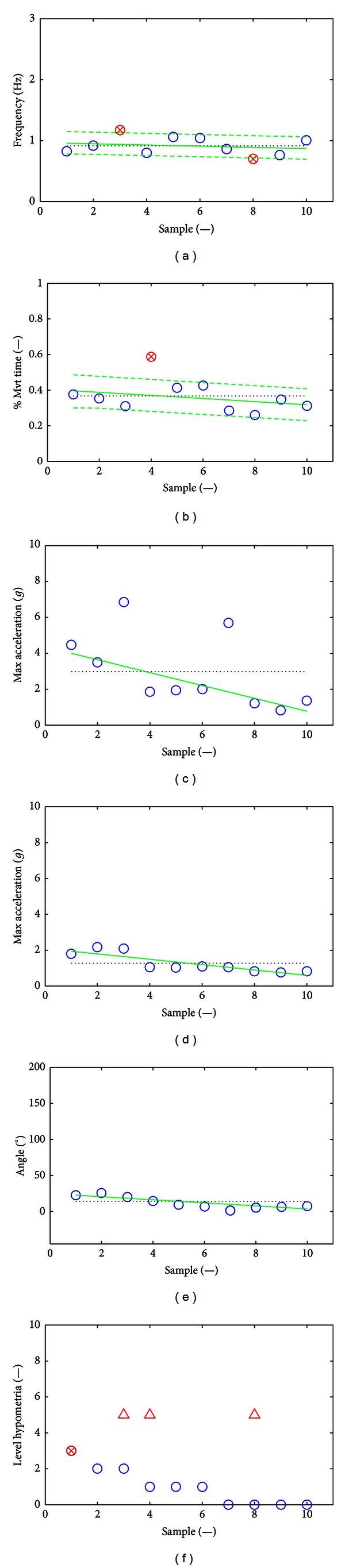
Typical features for an SMD consensus score of 3. Plot (a) gives the movement frequencies (circles). Plot (b) gives the percentages of movement time for maximum opening acceleration. Plot (c) gives the maximum closing accelerations. Plot (d) gives the maximum opening accelerations. Plot (e) gives the opening angles. Plot (f) gives the level of hypometria (circles) and the possible presence of initiation hesitations (triangles). For each plot except (f), the mean is in dotted line, the linear regression among points is in continuous line, and the limits for the detection of hesitations are in dashed lines (plots (a) and (b) only). The PD patient performed the FT task at a slower frequency, but there was no sign of decrementing or augmenting frequency. Maximum closing acceleration strongly decreases over samples, as for the maximum opening acceleration, which reflects a decrementing performance of the FT movement. Compared to the healthy volunteer, the mean values are lower, suggesting more difficulties in performing the movement. The mean opening angle is quite lower and decrements over time, reflected by a *Dangle* feature value of 2. So, the patient managed to keep a constant frequency but performed smaller and smaller movements. The patient does not present signs of hypometria. Three hesitations but no halts are detected. According to the analysis of the movement frequencies, the 3rd movement is abnormally fast, reflecting a jerky movement, the 8th movement is too slow, reflecting a hesitation while closing the fingers (lower value of percentage time for maximum opening acceleration), and the 4th movement is detected as an opening hesitation because the maximum opening acceleration occurs too late in the movement. On the frequency plot, this last movement is among the slowest.

After signal epoching, 18 features were extracted from the MDS-UPDRS FT task on the basis of the computed movement frequency, percentage of movement time for maximum opening acceleration, maximum closing and opening accelerations, opening angle, and *level of hypometria* as described in Section 2.3. The minimum and maximum values obtained for each feature across the 86 observations appear in Table 2. The raw signal and extracted features are presented for a SMD consensus score of 0 obtained from a healthy volunteer (Figures 2 and 7) and an SMD consensus score of 3 obtained from a PD patient (Figures 3 and 8). The healthy volunteer showed steady performance over repetitive FT movements. The movement amplitude is big and constant. The PD patient performed the FT task at a slower frequency but there was no sign of decrementing or augmenting frequency. The mean opening angle is quite lower and decrements over time, reflected by a *Dangle* feature value of 2. So, the patient managed to keep a constant frequency, but performed smaller and smaller movements. Three hesitations are also detected. These interpretations of movement features have been validated by examination of the corresponding video recordings.

### 3.3. Ordinal Logistic Regression Model and Feature Selection

**Figure 9:**
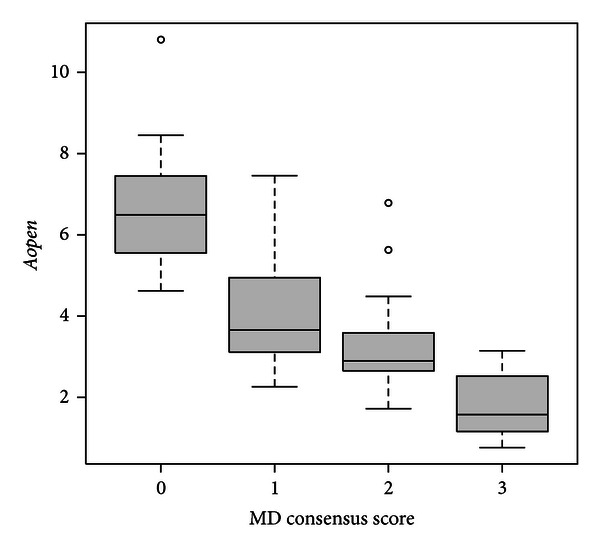
Plot of *Aopen* values according to SMD consensus scores. An increase of *Aopen* tends to conduct to a lower SMD consensus score, as reflected by the negative *β*_11_ coefficient.

Feature selection was performed on the 86 observation in order to build the global model. This model included 12 features as predictor variables. The model parameter estimates appear in Table 3. The *α* parameters have no particular interpretation. The positive (negative) *β* parameters correspond to variables that are positively (negatively) correlated with the SMD consensus scores. For example, as the *β*_11_ coefficient is negative, an increase of *Aopen* tends to conduct to a lower SMD consensus score. This is illustrated in Figure 9. The absolute value of these parameters have to be carefully interpreted by taking into account the range of the corresponding variables. The very high *β*_9_ coefficient is partially due to the very small range (0.063) of *slTopen*. As all observations were used to train this model, its performance could not be evaluated on an independent test dataset. Nevertheless, it was estimated with a cross-validation strategy.

### 3.4. Predictive Performance Evaluation

After the nested (inner and outer loop) cross-validation, each observation was associated with a prediction score. As these scores are on a continuous scale between 0 and 3, thresholds must be defined in order to classify the observations in the different ordered classes and compute some of the performance indexes. As a first approximation, thresholds of 0.5, 1.5, and 2.5 were used. A Goodman-Kruskal Gamma index of 0.961 was obtained between the predictions and the SMD consensus scores. The ordered contingency table (Table 4) indicates the joint frequency distribution of both the predictions and the SMD consensus scores. We observed that the maximum deviation between the predictions and the SMD consensus scores is 1 and that most observations are on the diagonal. 

**Figure 10:**
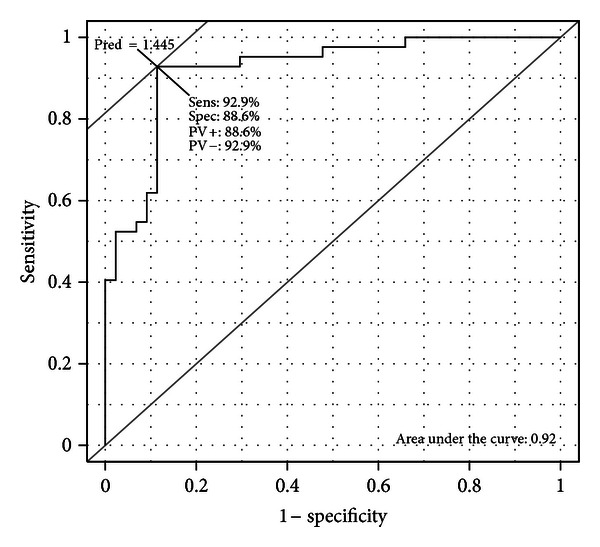
ROC curve obtained for the second classification task. ROC curves are used to optimize sensitivity and specificity and to compute AUC.

The sensitivity, the specificity, the accuracy, and the AUC of the ROC were computed using the predictions obtained with the nested cross-validation for each binary classification task and appear in Table 5. The ROC curve obtained for the second classification task is presented in Figure 10.

During the nested cross-validation, feature selection was performed 86 times, that is, one time for each iteration of the outer loop.  Table 6 summarizes the frequency of selection for each variable. The eight variables (*Dfreq*, *Afreq*, *Hesits*, *Halts*, *slTopen*, *slAclose*, *Aopen,* sand *slAopen*) selected as relevant in most iterations (at least 90% of the time) are presented in bold in Table 6. These features are all among the features selected during the construction of the global model presented in Section 3.3. 

## 4. Discussion

We have presented here a new system designed to predict MDS-UPDRS scores on the basis of features extracted from signals recorded during the FT task. The FT task is commonly used to assess movement bradykinesia in PD patients. In this task, subjects are asked to repetitively tap their index finger on their thumb as quickly as possible. The first 10 movements are used for scoring.

The presented method was developed on data obtained from 10 healthy volunteers and 36 PD patients to create a set of 92 observations with a range of FT scores between 0 and 4—as rated by three SMDs—according to the MDS-UPDRS criteria (Table 1). However, patients with a score of 4 at the MDS-UPDRS FT task were immediately detected as so by the system as they were not able to perform the task. It was neither possible nor necessary to include them in subsequent analyses. Removing those 6 observations led to a set of 86 observations used for further analyses. The healthy volunteer/PD patient status and the on/off medication status were not relevant for the purpose of this study.

An SMD consensus score has been defined for each observation by taking the average score of three SMD rounded to the nearest integer. There was a good level of agreement between the individual scores given by the three SMDs and the SMDs consensus scores, as reflected by the Goodman-Kruskal Gamma indexes (0.922, 0.982, and 0.992). However, all SMDs were not always in agreement with each other, as reflected by some of the Goodman-Kruskal Gamma indexes obtained between the individual scores (0.870), which confirms the need of a SMD consensus score. We acknowledge that a higher number of SMD may help refining SMD consensus scores.

The raw accelerometer signals were first epoched automatically to decompose the accelerometer signals into successive single FT movements. The success rate of this epoching was 99%. However, two parameters had to be adapted manually for tremulous PD patients and for those with a score of 3, who typically have hesitations or halts in movements. This problem could be eventually addressed by including contact switches to demonstrate a physical contact between the index and the thumb fingertips during each trial. However, using this approach, some adjustment would still be required for trial identification when the patient has difficulties in splitting fingers apart (i.e., freezing). Therefore, while the current system can identify individual finger movements with a 99% accuracy in the population under consideration, we acknowledge that achieving a 100% accuracy would require additional development both at the hardware and software levels. This is a necessary step to make this tool available for daily clinical practices. *z*-axis raw signals and their epoching, obtained for SMD consensus scores of 0 and 3, are presented in Figures 2 and 3, respectively.

Eighteen FT task movement features have been defined and extracted on the epoched acceleration signals to capture most of the FT movement characteristics. Among those features, eight features were based on MDS-UPDRS clinical characteristics and ten were accelerometer-specific features that cannot be easily assessed by visual inspection. The minimum and maximum values obtained for each feature across the 86 observations are presented in Table 2. Figures 7 and 8 illustrate the features obtained for SMD consensus scores of 0 and 3, respectively.

The main goal was to develop a tool to predict objective MDS-UPDRS scores from FT task movement features and to identify which of the eighteen features best predicted MDS-UPDRS FT scores given independently by the three SMDs on the basis of the corresponding video recordings. Since the global model was trained on all the observations, its predictive performance could not be evaluated. In order to estimate it, a leave-one-out cross-validation approach was used and a prediction score was obtained for each of the 86 observations. The predictive performance of the global model was estimated by comparing these continuous predictions to the SMD consensus scores. In order to compute some of the performance indexes, the continuous predictions were discretized using thresholds (0.5, 1.5, and 2.5). These discretized scores were first used to compute the Goodman-Kruskal Gamma index that tests the strength of association between the SMD consensus scores and the prediction scores. A value of 0.961 was obtained, which is similar to the ones obtained between the individuals scores given by SMD, that ranged from 0.870 to 0.970. The ordered contingency table between SMD consensus scores and score predictions is presented in Table 4. Most observations are on the diagonal, which corresponds to the same value for both the prediction and the SMD consensus score. Moreover, the maximum deviation between the predictions and the SMD consensus scores is 1 meaning that a patient with an SMD consensus score of 2 was sometimes misclassified in class 1 or 3 but never in class 0.

In order to compute the sensitivity, the specificity, the accuracy, and the AUC of the ROC, the ordinal classification problem was redefined into three binary classification problems. An ROC curve as well as its AUC were computed for each binary classification. Figure 10 presents the ROC curve obtained for the second classification task (i.e., to distinguish between FT scores 0-1 and 2-3). AUCs between 0.919 and 0.970 were obtained. It means that an observation with a given SMD consensus score will obtain a higher predicted score than observations associated with lower SMD consensus scores in most cases (>92%). Sensitivity and specificity were computed from the discretized scores for the three binary classification problems. In a future work, these thresholds could be optimized by including more observations. The identification of these thresholds can indeed be performed on smooth ROC curves obtained for numerous observations, by optimizing the sensitivity and the specificity. These thresholds could also be chosen by taking the “cost” of the different misclassifications into account. Including more observations will also probably increase the predictive performance of the model. However, in this paper, the goal was to show that this technique is adapted and works for the prediction of MDS-UPDRS scores. Future work will increase the number of observations to improve the predictive performance of the model.

During the cross-validation, feature selection was performed 86 times. The features that were selected as relevant in most iterations (Table 6) are all among the ones chosen to build the global model. Some of these features are the same as those proposed in the MDS-UPDRS, as *Dfreq*, *Afreq*, *Hesits,* and *Halts*. According to the instructions, SMDs have to quantify a possible slowing on the basis of a visual analysis of the patient's motor performance, for example. The *Dfreq* feature describes this possible slowing and can, in addition, give the index where the slowing occurs. *slTopen*, *slAclose*, *Aopen,* and *slAopen* are accelerometer-specific features that cannot be easily quantified by visual inspection. The possible decrementing amplitude (*Dangle*) has not been selected as a relevant feature for score prediction while it is one of the three main criteria that should be considered for scoring according to the MDS-UPDRS scoring instructions (Table 1). To better interpret this finding, it would have been useful to assess how often the amplitude decrement criterion was used by SMD to score the FT task. The data available here does not allow testing for this. Indeed, the score given by SMD was based on the presence of any of the three criteria, but SMDs were not asked to report which criteria was (were) selected for each given score. Thus, it is conceivable that the weight of the decrementing amplitude criteria for FT scoring is relatively low for both SMD and the proposed algorithm. The alternative explanation is that this feature is differentially considered by SMD and the computer-based method. In this case, one should consider inadequate computation in case of tremor or too fast movements or that it may be represented inside another accelerometer-specific feature such as *slAopen* since the intrinsic nature of the accelerometer measurements is different from the one of the human eye. It is not because the prediction algorithm does not use the decrementing angle as a predictive variable that this feature should not be used by MDs during their assessment. The accelerometer-based device is possibly not able to represent this feature in a way that can help to predict the UPDRS score. This is why other features are also used, as they may have a greater intrinsic ability to represent motor features used to predict MDS-UPDRS scores. The difference between the MDS-UPDRS features and those selected by the model can therefore be explained. First, the system allows the quantification of features that cannot be detected by visual analysis where; on the other hand, visual analysis allows taking into account the whole movement while accelerometers only summarize it into eighteen features. Second, the statistical predictive model objectively combines all the relevant features while it is difficult to simultaneously focus on all movement features by simple visual inspection.

Since the developed tool is low-cost, easy-to-use in daily clinical practices and as it shows very good predictive performance, it may be used as a support decision tool. The other tasks of the MDS-UPDRS can easily be integrated in the tool since only the feature extraction algorithm has to be adapted for each task while the model is automatically computed based on the new data sets. For every new patient performing an MDS-UPDRS task, the relevant features would be extracted using the observations returned by the tool. The ordinal logistic regression model would then compute an MDS-UPDRS score from the values of these features. Since the prediction of the model is continuous, it is more sensitive than a 5-point scoring system. It could therefore be a valuable asset to assess the evolution of the disease and treatment efficacy and could help SMD take a decision in ambiguous cases.

## Figures and Tables

**Table 1:** MDS-UPDRS instructions for FT task scoring [4] (instruct the patient to tap the index finger on the thumb 10 times as quickly and as big as possible. Rate each side separately, evaluating speed, amplitude, hesitations, halts, and decrementing amplitude).

Score	Scoring instructions

0	No problems

1	Any of the following:
	(a) the regular rhythm is broken with one or two interruptions or hesitations of the tapping movement;
	(b) slight slowing;
	(c) the amplitude decrements near the end of the 10 taps.

2	Any of the following:
	(a) 3 to 5 interruptions during tapping;
	(b) mild slowing;
	(c) the amplitude decrements midway in the 10-tap sequence.

3	Any of the following:
	(a) more than 5 interruptions during tapping or at least one longer arrest (freeze) in ongoing movement;
	(b) moderate slowing;
	(c) the amplitude decrements starting after the 1st tap.

4	Cannot or can only barely perform the task because of slowing, interruptions, or decrements.

**Table 2:** Features summary with minimum and maximum values across the 86 observations. The 8 MDS-UPDRS features are presented first.

Feature	Feature definition	Min	Max
*Freq *	Mean movement frequency (Hz)	0.367	5.106
*Dfreq *	Index for decrementing frequency (—)	2	10
*Afreq *	Index for augmenting frequency (—)	2	10
*Angle *	Mean opening angle (°)	5.139	117.8
*Dangle *	Index for decrementing angle (—)	2	10
*Hypom *	Hypometria (—)	0	10
*Hesits *	Number of hesitations (—)	0	5
*Halts *	Number of halts (—)	0	1
*Topen *	Mean, standard deviation, and slope of percentage of movement time for maximum opening acceleration (—)	0.191	0.533
*sdTopen *		0.017	0.232
*slTopen *		−0.031	0.032
*Aclose *	Mean, standard deviation, and slope of maximum closing acceleration (g)	1.131	9.676
*sdAclose *		0.338	3.145
*slAclose *		− 0.448	0.603
*Aopen *	Mean, standard deviation, and slope of maximum opening acceleration (g)	0.761	10.81
*sdAopen *		0.054	1.483
*slAopen *		−0.304	0.364
*RMS *	Root mean square (g)	0.942	4.047

**Table 3:** Model parameter estimates.

Parameter	Estimation
*α*_0_	− 18.768
*α*_1_	− 11.989
*α*_2_	− 4.066
*β*_1_ (*Freq*)	− 0.647
*β*_2_ (*Dfreq*)	− 0.726
*β*_3_ (*Afreq*)	− 0.238
*β*_4_ (*Dangle*)	− 0.411
*β*_5_ (*Hesits*)	1.398
*β*_6_ (*Halts*)	16.653
*β*_7_ (*Topen*)	5.858
*β*_8_ (*sdTopen*)	25.089
*β*_9_ (*slTopen*)	112.52
*β*_10_ (*slAclose*)	3.966
*β*_11_ (*Aopen*)	− 1.171
*β*_12_ (*slAopen*)	− 9.711

**Table 4:** SMD consensus scores versus predicted scores.

		SMD consensus scores			
Predicted scores		** 0 **	** 1 **	** 2 **	** 3 **
	** 0 **	9	3	0	0
	** 1 **	3	24	6	0
	** 2 **	0	5	24	2
	** 3 **	0	0	1	9

**Table 5:** AUC of the ROC, sensitivity, specificity, and accuracy for each binary classification task.

Groups	AUC	Sens.	Spec.	Acc.
0 versus 123	0.945	0.750	0.959	0.930
01 versus 23	0.919	0.886	0.900	0.872
012 versus 3	0.970	0.986	0.818	0.965

**Table 6:** Number of selection of each feature during the leave-one-out cross-validation.

Nb	Feature	Occur.
1	*Freq *	72
2	***Dfreq ***	**84**
3	***Afreq ***	**83**
4	*Angle *	53
5	*Dangle *	64
6	*Hypom *	49
7	***Hesits ***	**83**
8	***Halts ***	**86**
9	*Topen *	49
10	*sdTopen *	54
11	***slTopen ***	**85**
12	*Aclose *	18
13	*sdAclose *	27
14	***slAclose ***	**86**
15	***Aopen ***	**83**
16	*sdAopen *	29
17	***slAopen ***	**78**
18	*RMS *	20
